# BayesPI-BAR2: A New Python Package for Predicting Functional Non-coding Mutations in Cancer Patient Cohorts

**DOI:** 10.3389/fgene.2019.00282

**Published:** 2019-04-02

**Authors:** Kirill Batmanov, Jan Delabie, Junbai Wang

**Affiliations:** ^1^Department of Pathology, Norwegian Radium Hospital, Oslo University Hospital, Oslo, Norway; ^2^Department of Pathology, University Health Network, Toronto, ON, Canada

**Keywords:** gene regulation, transcription factors, cancer, bioinformatics, non-coding mutations

## Abstract

Most of somatic mutations in cancer occur outside of gene coding regions. These mutations may disrupt the gene regulation by affecting protein-DNA interaction. A study of these disruptions is important in understanding tumorigenesis. However, current computational tools process DNA sequence variants individually, when predicting the effect on protein-DNA binding. Thus, it is a daunting task to identify functional regulatory disturbances among thousands of mutations in a patient. Previously, we have reported and validated a pipeline for identifying functional non-coding somatic mutations in cancer patient cohorts, by integrating diverse information such as gene expression, spatial distribution of the mutations, and a biophysical model for estimating protein binding affinity. Here, we present a new user-friendly Python package BayesPI-BAR2 based on the proposed pipeline for integrative whole-genome sequence analysis. This may be the first prediction package that considers information from both multiple mutations and multiple patients. It is evaluated in follicular lymphoma and skin cancer patients, by focusing on sequence variants in gene promoter regions. BayesPI-BAR2 is a useful tool for predicting functional non-coding mutations in whole genome sequencing data: it allows identification of novel transcription factors (TFs) whose binding is altered by non-coding mutations in cancer. BayesPI-BAR2 program can analyze multiple datasets of genome-wide mutations at once and generate concise, easily interpretable reports for potentially affected gene regulatory sites. The package is freely available at http://folk.uio.no/junbaiw/BayesPI-BAR2/.

## Introduction

Somatic mutations are the primary cause of cancer. Although most studies of cancer genomes to date have focused on mutations occurring within exons, recent efforts have made whole genome sequences of paired tumor and normal samples widely available, facilitating the analysis of non-coding variants in cancer. In many cases, such variants have been shown to affect gene expression and to promote tumorigenesis (Khurana et al., 2016). One mechanism by which non-coding variants can affect gene expression is the alteration of TF binding to mutated DNA sequences. For example, a mutation may disrupt a TF binding site, preventing the TF from recognizing its target sequence, or a new binding site may be created by a mutation. Several computational tools are available to predict such effects, e.g., GERV (Zeng et al., 2016), atSNP (Zuo et al., 2015), BayesPI-BAR (Wang and Batmanov, 2015), among others. All these tools have the same mode of operation: given a mutation, typically a SNV, and a set of TF-DNA binding models, they produce a list of TFs whose binding is possibly affected by the SNV, ordered by the effect size and/or certainty. However, the predicted list may contain dozens of TFs for every SNV. Adding to the complexity of issue, each cancer sample may have thousands of SNVs, which makes it difficult to interpret the results. Importantly, there is no software package available today to perform such analysis for a patient cohort based on genome-wide sequencing data, considering recurring effects of mutations among several patients.

The BayesPI-BAR2 package presented here aims to solve these problems. It ranks TFs affected by SNV through a new BayesPI-BAR algorithm (Batmanov et al., 2017), augmented with a set of tools to find mutation hotspots among patients and mutations linked to differentially expressed genes. The pipeline collects information about SNVs of all patients in the mutation hotspot regions, and then evaluates the significance of predicted effects against randomly generated background mutation models. The methodology behind BayesPI-BAR2 package and the robustness of predictions were validated in a previous study (Batmanov et al., 2017). Now, a user-friendly Python package is developed based on the proposed pipeline. The package is evaluated in both FL and skin cancer patients, by using mutations called from the whole genome sequencing experiments. BayesPI-BAR2 may reveal novel regulatory sites that are disrupted by mutations in cancer or other diseases, by using genome-wide sequencing data, which is similar to the findings in Weinhold et al. (2014). Additionally, it can identify novel TFs whose binding is altered by non-coding mutations in the genome (Batmanov et al., 2017). It is useful not only for regulatory mutation study in cancer, but also for similar research in other diseases.

## Materials and Methods

### Overview of BayesPI-BAR2 Python Package

**FIGURE 1:**
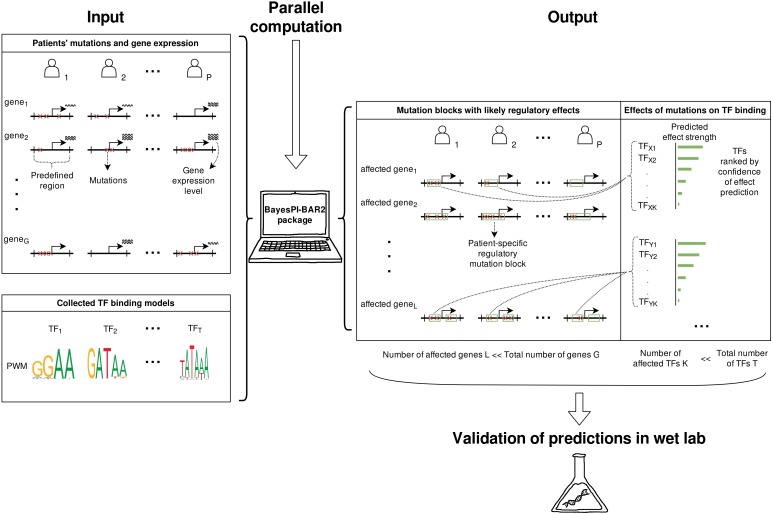
Overview of the BayesPI-BAR2 operation. Using patients’ mutation and the corresponding gene expression data, BayesPI-BAR2 finds mutation hotspots and patient-specific mutation blocks. Then, using a given set of PWMs for TFs of interest, it produces a list of significant TFs whose binding is potentially affected at mutation block. The predictions consider all available mutation data among patient samples, to which strict statistical tests are applied to determine the significance of predicted effects. As a result, the number of predicted effects is small enough for performing follow-up wet lab validation.

The operation of the BayesPI-BAR2 pipeline is illustrated in Figure 1. It is motivated by works in Batmanov et al. (2017) where novel mutations affecting gene regulation were discovered in FL patients, by considering diverse genome information. The original analysis pipeline comprised of various scripts that were implemented in different programming languages. Here, a completely new Python package was built with enhanced functionality and user-friendly command line options. Particularly, the old BayesPI-BAR (Wang and Batmanov, 2015) program (a combination of R and Perl programs) was reimplemented in Python with a more efficient algorithm and flexible parallelization. This computationally demanding task can be automatically parallelized now either on a single multi-core machine, or on a cluster supporting the SLURM job queue manager.

BayesPI-BAR2 Python package first finds DNA regions with high mutation density and close to differentially expressed genes, then predicts TF affinity changes in these regions using the new BayesPI-BAR, and finally tests the significance of these predicted changes against a background model. All analysis is carried out by a set of command line tools written in Python 2. The package also includes binary files of the new BayesPI program (Wang and Morigen, 2009) which can infer new TF binding affinity models PWMs such as dinucleotide interdependence (Wang, 2014), DNA shape-restricted dinucleotide models (Batmanov and Wang, 2017), and compute TF-DNA differential binding affinity (dbA) scores (Wang et al., 2015). There is also a demo script in the package that shows a full pipeline execution. BayesPI-BAR2 Python package is a useful tool for identifying functional regulatory mutations in cancers or diseases, based on whole genome sequencing experiments. For a more detailed description of the package, please refer to following sections and (Batmanov et al., 2017).

### Identification of Mutation Hot Regions and Patient-Specific Mutation Blocks

In the first step of the BayesPI-BAR2 pipeline, highly mutated DNA sequence (mutation hotspot) regions are identified by a method described in Batmanov et al. (2017), which considers mutations from several patients to define a set of regions. In default setting, BayesPI-BAR2 searches for putative mutation hotspot regions near the transcription start sites (TSS) of differentially expressed genes, because important regulatory sequences (e.g., functional regulatory mutations) are often located in the promoters. To have a robust mutation calling (Alioto et al., 2015) in the promoter region, a minimum sequencing depth of 30 is recommended at this point. The significance of the differential expressions is tested by two-sample Kolmogorov–Smirnov test, where *reads per kilobase of exon model per million mapped reads* (RPKM) values of RNA-seq data of patients are compared to that of the normal samples (e.g., *P* < 0.05). Since RPKM-based differential expression tests may be affected by experimental biases (Bullard et al., 2010) and result in imprecise prediction, a multiple testing correction of *P*-values is not recommended. Nevertheless, by changing the threshold value of the pipeline, it is easy to apply the Bonferroni correction on the *P*-values. Alternatively, user can apply external software to perform the differential gene expression analysis, and directly input the gene list into BayesPI-BAR2 package.

Subsequently, MuSSD (Mutation filtering based on the Space and Sample Distribution) algorithm (Batmanov et al., 2017) is applied on the promoter regions of differentially expressed genes. Based on the identified mutation hotspot regions from MuSSD, patient specific mutation blocks are built: the reference sequence is taken from the reference genome assembly according to the region covered by the mutation hotspot (possibly including patient germline variants), and the alternate sequence contains all mutations from the same patient in the region. In BayesPI-BAR2 package, the computational predictions of both the mutation hotspot regions and the patient-specific mutational blocks are implemented in Python, with a more efficient algorithm than the original MATLAB script (Batmanov et al., 2017).

### BayesPI TF-DNA Binding Affinity Model

The basic biophysical model for computing TF-DNA binding affinity, named BayesPI, was first reported in Wang and Morigen (2009). The TF-DNA binding probability is derived from the statistical mechanical theory of TF-DNA interactions (Djordjevic et al., 2003; Foat et al., 2006), which can be shown as

$$P\left(S,w,\mu \right)=\sum_{i=0}^{N-M}\frac{1}{1+e^{E_{\mathrm{indep}}\left(S_{i:i+M},w\right)-\mu }}$$

where *S*_i,a_ = 1 if the DNA sequence has nucleotide a (one of A, C, G, T) at position i and *S*_i,a_ = 1 otherwise, *N* is the sequence length, *M* is the length of the binding motif, μ is the chemical potential of the TF or its concentration in the nucleus. The selection of μ (e.g., μ = 0, -10, -13, -15, -18, -20) is based on a previous study (Wang and Batmanov, 2015) of the effect of DNA sequence variants on TF binding affinity changes, where verified regulatory mutations in human genome were used to infer the dynamical range of chemical potentials.

$$E_{\mathrm{indep}}\left(S,w\right)=\sum_{j=0}^{M-1}\sum_{a=1}^{4}w_{j,a}S_{j,a}$$

*E*_indep_ (*S, w*) is the TF binding energy to a short DNA fragment with length M bp. This model assumes that nucleotides at each binding position contribute to the binding energy independently. The matrix *w* ∈ *R*^(M×4)^, called position-specific affinity matrix (PSAM), where *w*_j,a_ is the binding energy of nucleotide a at position j of the DNA fragment. In BayesPI-BAR2 Python package, a collection of PSAMs derived from a previous published work (Kheradpour and Kellis, 2014) is included, and several new BayesPI features are also added [e.g., PSAM with dinucleotide interdependence (Wang, 2014), and DNA shape-restricted dinucleotide models (Batmanov and Wang, 2017)].

### BayesPI-BAR Approach

Bayesian modeling of Protein-DNA Interaction and Binding Affinity Ranking (Wang and Batmanov, 2015) method is used to evaluate the significance of TF binding affinity changes caused by DNA sequence variants. It is based on an idea for distinguishing direct versus indirect TF binding in Wang et al. (2015). A new quantity, dbA, is introduced to measure the binding strength above background level. BayesPI-BAR Python code computes the *shifted differential binding affinity* (δdbA), for each sequence variant and TF:

$$\delta dbA\left(S_{\mathrm{ref}},S_{\mathrm{alt}}\right)=dbA\left(S_{\mathrm{alt}}\right)-dbA\left(S_{\mathrm{ref}}\right)$$

*S*_ref_, *S*_alt_ represent the reference and alternate sequences, respectively. δdbA is the measure of the affinity change used by BayesPI-BAR. More details about the BayesPI-BAR approach are available in the supplementary and (Batmanov et al., 2017).

### Significance Testing for TF Binding Affinity Changes

To test the significance of disruption of TF-DNA binding by patient SNVs, patient-specific δdbA values of a given regulatory mutation block are compared to that of the randomly generated background mutation blocks, using the two-sided Rank-sum test. BayesPI-BAR2 has three alternative mutation models to generate the background: a tumor-derived mutation model, a k-mer *mutation signature* such as those available from COSMIC (Tate et al., 2018), and a uniform mutation model. A list of TF binding effects which are significantly stronger than estimated by the background model is exported by BayesPI-BAR2.

**FIGURE 2:**
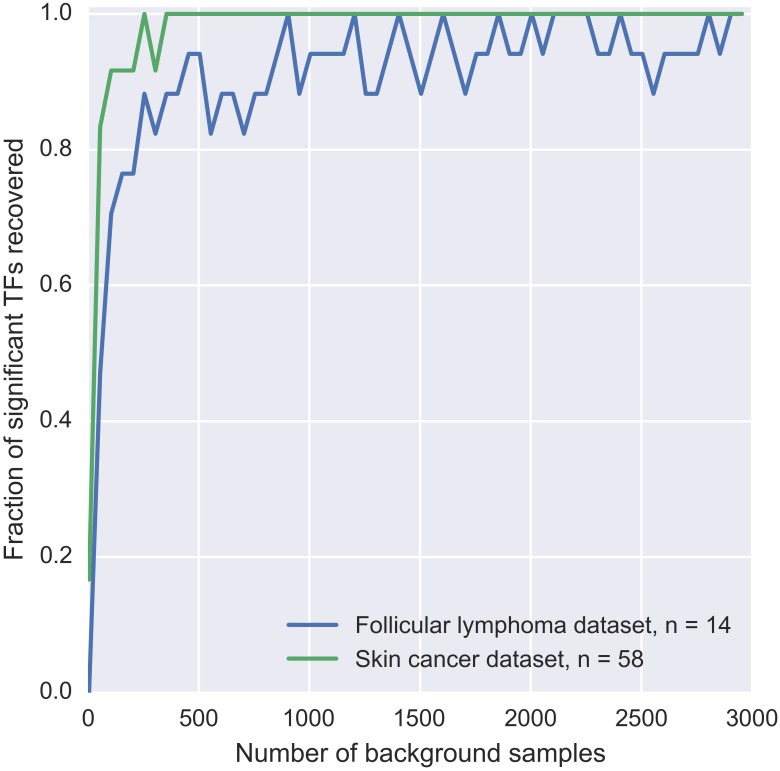
Estimation of sufficient background samples for BayesPI-BAR2 package. The plot displays the dependency of significant TF discovery on the number of background samples used. Significant TFs in the mutation blocks from two different datasets are considered: (1) two *BCL2* blocks from FL dataset with 14 patients affected, blue line; (2) and the *TERT* block from skin cancer dataset with 58 patients affected, green line. On the *X*-axis, we plot the number of background mutation blocks taken. On the *Y*-axis, we plot the number of significant TFs found when using X background mutation blocks, which are *also* significant when using the full set of 10000 background blocks. Y is normalized by the number of significant TFs discovered using the full background set. Therefore, *Y* = 1 corresponds to the same result as using the full background set.

Since patient mutation blocks are pre-filtered by MuSSD algorithm based on the space and sample distribution of mutations, there are several constraints on the background mutation blocks: (a) both the size and the mutation counts of the background mutation blocks are kept same as that of patient ones. (b) DNA sequence is selected randomly from the same regions as the patient mutation block. (c) distributions of the mutation positions and the nucleotide changes are based on specific mutation signature such as tumor-derived mutations. To evaluate the relationship between the number of background blocks and the precision of background δdbA model, a few simulations are displayed in Figure 2. It shows the fraction of significant TFs reaches a plateau when there are more than 1000 blocks used. The significance test for TF-DNA binding affinity changes proceeds in following three steps:

(1) Background mutation blocks are extracted randomly from regions of interest, with the same sequence length as patient block. Reference sequence of a background mutation block is taken from the reference genome. The alternate sequence is generated by random alteration of nucleotides in reference sequence, using either the tumor-derived mutations or the given k-mer mutation probability distribution (the mutation signature).
(2) For each given TF, BayesPI-BAR computes δdbA of a patient regulatory mutation block. Then, it computes δdbA values for about 2000 background blocks that represent the background distribution of δdbA scores.
(3) Wilcoxon rank-sum test is used to compare the distribution of δdbA values between the patients’ and the background mutation blocks. Bonferroni correction of *P*-values is applied.

The significance testing considers both the strength of TF binding affinity change and the recurrence of δdbA values across samples, using the Bonferroni correction for the number of TFs tested. A stronger *P*-value correction procedure may not be suitable here. For example, Benjamini–Hochberg (BH) false discovery rate requires the *P*-values to be independent (or have limited dependencies) (Benjamini and Hochberg, 1995; Benjamini and Yekutieli, 2001), but there are strong dependencies among *P*-values of the significance testing for TF binding affinity changes. Often, *P*-values of very similar PWMs are close to each other, which may result in unreliable correction by the BH procedure. Bonferroni correction has no assumptions about the process used to generate the *P*-values which is suited in the current study. At least 10 samples are needed to perform proper statistical test in BayesPI-BAR2. If the sample size is too small, there will be a problem in achieving the statistical significance by Rank-sum test, even if the effects are large (Wild and Seber, 2011).

### Algorithm Efficiency and Parallel Computation

Computation of scores is the most time-consuming task that is needed for both the patient and the background mutation blocks. The old R program (Wang and Batmanov, 2015) was designed to evaluate TF binding affinity changes in a single mutation and was unable to process multiple mutations simultaneously. In the new Python package, a parallel computation paradigm is developed by using more efficient data processing library. Additionally, the efficiency of BayesPI code was improved by applying a new sub-expression for TF binding probability (please refer to BayesPI TF-DNA binding affinity model section):

$$e^{\sum_{j=0}^{M-1}\sum_{a=1}^{4}w_{\mathrm{j,a}}S_{\mathrm{j,a}}-\mu }=e^{-\mu }\prod_{j=0}^{M-1}\prod_{a=1}^{4}{\left(e^{w_{\mathrm{j,a}}}\right)}^{S_{\mathrm{j,a}}}$$

Where the terms e^w_j,a_^ and e^-μ^ in the right side of the formula are precomputed and stored in order to avoid computing the exponent term in every sliding window. The new implementation reduces the computational time by about 90%. In addition, in BayesPI-BAR2 Python package, all calculations are parallelized across either multiple local CPUs or multiple nodes on a cluster using the SLURM workload manager. For instance, it takes about 5 h to process all mutation blocks in the skin cancer dataset (263 patients; ∼100000 mutations), by using 8 nodes of 8 CPUs in each. The overall waiting time can be further reduced if more parallel processes are used or few mutation blocks are selected for testing. User guide and package architecture of BayesPI-BAR2 are available in the Supplementary Section.

## Results

### Validating New Python Code in Verified Regulatory Mutations

The precision of the new BayesPI-BAR Python program, which is the basis of BayesPI-BAR2 package, was first assessed by a benchmark dataset of 67 SNVs with experimentally verified effects of TF binding. The results match the previous study (Wang and Batmanov, 2015).

### Evaluating the New BayesPI-BAR2 Package in Follicular Lymphoma

A previous analysis of regulatory mutations in FL cancer patients was performed by running various scripts manually. The new BayesPI-BAR2 Python package is applied on the same FL patients, by considering only the gene promoter regions (e.g., TSS ± 1000 bp with 795 called SNVs) as were investigated before (Batmanov et al., 2017). Putative mutation hot blocks near *BCL6, BCL2*, and *HIST1H2BM* genes are detected automatically, where containing 34, 40, and 2 SNVs, respectively. The results match with the earlier report (Batmanov et al., 2017). Also, the mutation effects on TF binding at the promoter of two important FL genes (*BCL6* and *BCL2*) (Pasqualucci et al., 2014) were recovered: for example, regulatory activities of two TFs (*FOXD2* and *FOXD3*) on *BCL6* and *BCL2* were confirmed previously by knockdown experiments in SUDHL4 lymphoma cell (Batmanov et al., 2017). The new BayesPI-BAR2 Python package can reproduce the previous results (Batmanov et al., 2017) and is robust in predicting functional regulatory mutations.

### Applying BayesPI-BAR2 on Genome-Wide Sequencing Data of Skin Cancer

The somatic mutations and RNA-Seq counts for the skin cancer evaluation were downloaded from the public DCC data release 23 at the International Cancer Genome Consortium (ICGC) data portal, from the MELA-AU, SKCA-BR, and SKCM-US projects. The dataset contains 23 million mutations called from whole genome sequence analysis of 263 patients. Melanoma or skin cancer has the highest prevalence of somatic mutations across human cancer types, which is more than ten times higher than that in Lymphoma cancer (Alexandrov et al., 2013). There are frequent driver coding mutations in melanoma cancer (Hodis et al., 2012; Roberts and Gordenin, 2014). Therefore, DNA regions from 2 Kbp upstream to 100 bp downstream of TSS of protein-coding genes [e.g., GENCODE (Harrow et al., 2012)] were selected, and genes differentially expressed between the patient RNA-Seq data and the normal melanocyte RNA-Seq (Haltaufderhyde and Oancea, 2014) were used in this study (10015 genes with ∼99173 mutations).

**FIGURE 3:**
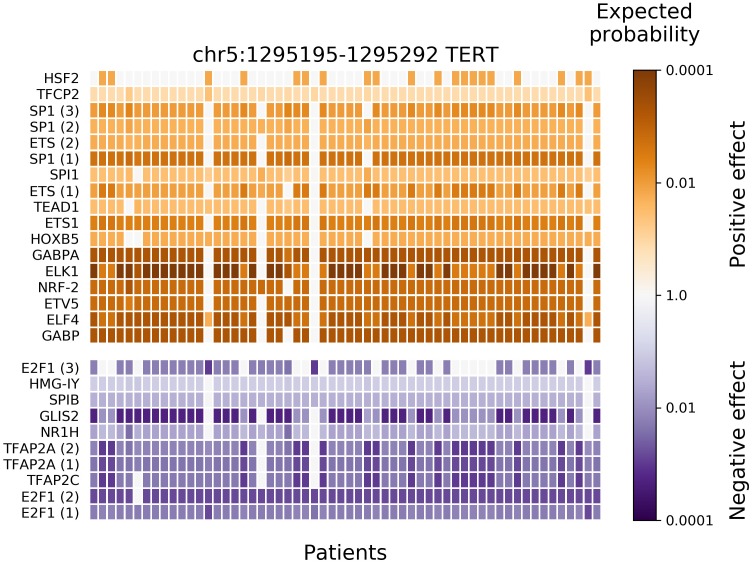
Results of new BayesPI-BAR2 package – TFs significantly affected by skin cancer somatic mutations in the *TERT* promoter mutation block. The heatmap displays the distribution of predicted TF binding effects of *TERT* promoter somatic SNVs across 58 skin cancer patients. The columns represent patients, the rows represent predicted significantly affected TFs, and the color represents the binding effect size. Reduced binding is shown in blue and increased binding in orange. The color shade represents the log_10_-scaled fraction of background δdbA values which are more extreme than the observed δdbA of a patient, which is an indication of effect size. The darker cells the larger effect. Some TFs are represented by multiple PWM models, their instances are indicated by a number in parentheses. Only significantly affected TFs (Wilcoxon rank-sum test *P*-value < 0.001 after Bonferroni correction) are shown. The following TFs belong to the ETS family (Gutierrez-Hartmann et al., 2007): GABPα, ELF4, ETV5, ELK1, ETS1, SPI1, and SPIB. Here, background mutation model in BayesPI-BAR2 is based on tumor samples.

After applying BayesPI-BAR2 Python package, 166 putative regulatory mutation blocks were detected (containing 2746 mutations). A list of the 15 most highly mutated blocks is shown in a Supplementary Table 1, where blocks matched to previous findings are marked and the corresponding publications are cited. A mutation block near *TERT* gene has the most patients affected, 58 in number, closely followed by blocks near several housekeeping genes (*RPL^∗^, RPS^∗^*, and others). This is in agreement with the previous studies (Weinhold et al., 2014; Poulos et al., 2015). It has been suggested that these mutations are due to vulnerability of some DNA positions to ultraviolet light damage (Fredriksson et al., 2017). In the *TERT* mutation block, significantly affected TFs were also predicted by BayesPI-BAR2 automatically (e.g., Wilcoxon rank-sum test *P* < 0.001 with Bonferroni correction; Figure 3), which split into two groups: positive change (creation of binding sites) at the top, in orange; and negative change (destruction of existing binding sites) on the bottom, in blue. The heatmap of Figure 3 shows the variation of affinity changes among 58 patients, who harbor at least one mutation in the *TERT* block. Nine out of seventeen positively affected TFs belong to the ETS protein family, which are the most significantly affected ones. This is also in agreement with the well-known pathomechanisms of melanoma (Huang et al., 2013). When testing significance of affinity changes against the skin cancer specific mutation signature model and a uniform model, the same significantly affected TFs were found in the *TERT* block, with small differences in the ranking (Supplementary Figures 1, 2).

Additionally, BayesPI-BAR2 discovers novel regulatory mutations which affect gene expression in skin cancer. For instance, binding of TFs from Sp/KLF family and ETS family were found to be disrupted (e.g., about 47 patients with mutations; Supplementary Table 1) in a mutation block near RALY. RALY is differentially expressed between the skin cancer patients and the normal control samples. It is an RNA-binding protein that may play a role in pre-mRNA splicing. Based on human phenotype association evidence for RALY from the GWAS Catalog (MacArthur et al., 2017), we found mutations of this gene associated with melanoma, skin pigmentation, and skin sensitivity to sun. The next most frequent mutation block was predicted near RPS27 (e.g., 46 patients with mutations), where binding of TBP, ETS, and IRF TF families are interrupted. RPS27 mutation and its elevated expression have been detected in many melanoma patients and in various human cancers (Dutton-Regester et al., 2014). The two newly discovered regulatory mutation blocks may contribute to the dysregulation of RALY and RPS27 and are worthy for further investigation because both genes are known to be significantly associated with melanoma. Thus, BayesPI-BAR2 not only can automatically recover known gene regulatory disturbance, but also can discover the novel ones which can be tested in wet-lab. BayesPI-BAR2 Python package comes with the code to perform the complete analysis of this melanoma dataset.

## Discussion and Conclusion

The new BayesPI-BAR2 Python package has been evaluated in both small (e.g., 14 FL patients) and large (e.g., 263 skin cancer patients) cancer patient cohorts, based on whole genome sequencing experiments. It achieves good prediction accuracy and automatically reproduces the published results. The new package can be used to investigate previously unknown regulatory effects, even if the sample size is small and the recurrent mutation frequency is low. Nevertheless, the robustness of significance test in BayesPI-BAR2 is dependent on the sample size (Biau et al., 2008), a small sample size may pose difficulty in achieving the significance difference. For example, there are 3 mutation blocks from 14 FL patients that pass the test of significant TF binding affinity changes (*P*-values <0.05), but there are 15 mutation blocks from 263 skin cancer samples that pass a more stringent criteria (*P*-values <0.001). Therefore, a large sample size is preferred when using BayesPI-BAR2 to predict putative functional non-coding mutations.

BayesPI-BAR2 approach is more general than a previous mutation recurrence analysis (Weinhold et al., 2014), because it takes into account the recurrence of both the mutation among multiple patients and the effect on TF binding. In other words, different mutations may contribute to the creation or disruption of the same regulatory link in different patients. For example, there are two canonical highly recurrent mutations in the *TERT* promoter mutations: C > T at chr5:1,295,228 and chr5:1,295,250. Both of these mutations create ETS binging sites. Though six of fifty-eight patients did not have these two mutations, some ETS factors are positively affected in five of them (Figure 3). It indicates that other non-canonical mutations at TERT promoter may also create ETS binding sites.

Although BayesPI-BAR2 needs heavy computation to achieve the goal, the waiting time can be significantly reduced by distributing more jobs in a high performance computing system. In the study of 263 skin cancer patients, the total waiting time was reduced to 1 h and 30 min while using 10 nodes of 10 CPUs of ABEL computer cluster at University of Oslo. On average, approximately 6 min are used for completing the calculation of one mutation block. Efficiency of BayesPI-BAR2 can be further improved by applying advanced sampling method and parallel algorithm, or by implementing it in Graphical Processing unit (GPU) (Zou et al., 2018). Alternatively, if more prior information regarding mutation blocks (e.g., differential methylation, nucleosome occupancy, active enhancer/promoter histone markers, and predicted long distance gene regulations) (Wang et al., 2013; Cao et al., 2017; Dhingra et al., 2017) is available, then fewer mutation blocks will be selected for testing against the background models. Thus additional information can also reduce the total computation time significantly. The new features will be implemented in the future.

The new BayesPI-BAR2 Python package allows analysis of non-coding mutations in cancer patient cohorts, discovering mutation hotspots, and predicting effects of these mutations on TF-DNA binding. Unlike previously available tools, it considers the frequency of mutations, their recurrence across patients, and integrates this information with the predicted affinity changes employing a simple and statistically sound approach. Although in principle, it is applicable to any mutation dataset, BayesPI-BAR2 is designed for the typical cancer use case, with the goal to find few non-random effects among many somatic mutations. The package can be a useful tool for in-depth analysis of non-coding mutations detected in whole genome sequencing experiments, as well as for predicting their effects on genome regulation in cancer. All in all, it provides a reasonable number of predictions for further experimental validation.

## Data Availability

The package source code, binaries for Linux and OS X, and demo datasets are available at http://folk.uio.no/junbaiw/BayesPI-BAR2/; Project name: BayesPI-BAR2 Package; Operating system(s): Linux and OS X; Programming language: Python; License: General Public License (GNU GPLv3); Any restrictions to use by non-academics: None; The datasets analyzed during the current study are available in the public DCC data release 23 at the ICGC data portal: https://dcc.icgc.org/releases/release_23/Projects.

## Author Contributions

KB implemented the BayesPI-BAR2 pipeline in Python. JD validated study. JW conceived project, designed BayesPI-BAR2 pipeline, and contributed in developing package. KB and JW drafted manuscript. All authors read and approved the final manuscript.

## Conflict of Interest Statement

The authors declare that the research was conducted in the absence of any commercial or financial relationships that could be construed as a potential conflict of interest.

## Abbreviations

BayesPI-BAR: Bayesian modeling of Protein-DNA Interaction and Binding Affinity Ranking
FL: follicular lymphoma
PWM: position weight matrix
SNV: single nucleotide variant
TF: transcription factor
