# Supplementary material for: BayesPI-BAR2: A New Python Package for Predicting Functional Non-coding Mutations in Cancer Patient Cohorts
Source: Front Genet. 2019 Apr 2;10:282. doi: 10.3389/fgene.2019.00282 (PMC6454009; doi:10.3389/fgene.2019.00282)
Supplement: Supplementary file 2 [file Table_2.docx]

**Supplementary information of "BayesPI-BAR2: a new Python package for predicting functional non-coding mutations in cancer patient cohorts"**

Kirill Batmanov, Jan Delabie, and Junbai Wang

**Supplementary Methods**

**1. Detailed description of BayesPI-BAR approach**

BayesPI-BAR (Wang and Batmanov, 2015) [method is used to evaluate the significance of TF binding affinity changes caused due to DNA sequence variants. It is based on an idea of earlier work](#_ENREF_3) (Wang et al., 2015) [for distinguishing direct versus indirect TF binding. There are two new quantities in the calculation: the](#_ENREF_4) *differential binding affinity* ($dbA$) and its corresponding P-value ($P_{dbA}$):

$dbA\left( S,R \right)=P\left( S \right)-\frac{1}{R}\sum_{i=1}^{R} P\left( \text{shuffle}\left( S \right) \right)$

$P_{dbA}\left( S,R \right)=\frac{1}{R}\text{count}_{R}\left[ P\left( S \right)>P\left( \text{shuffle}\left( S \right) \right) \right]$

Here $R$ is the number of randomly shuffled samples, $P\left( S \right)$ is the TF-DNA binding probability that depends on $w$ and $\mu$. $\text{shuffle}\left( S \right)$ is a function that randomly shuffles nucleotides in a sequence, and $\text{count}_{R}\left[ \right]$ is a function that counts how many times an argument is true in $R$ trials. It is assumed that the smaller the $P_{dbA}\left( S_{1},R \right)$ the stronger the TF-DNA binding to sequence $S_{1}$. The $dbA\left( S_{1},R \right)$ measures the strength of a TF-DNA binding when it is compared to that of the background model. $R$ controls the precision of estimations, which increases with increasing $R$. Usually, $R=10000$ gives sufficient accuracy for the prediction.

BayesPI-BAR computes an additional score, *shifted differential binding affinity* ($\delta dbA$), for each sequence variant and TF:

$\delta dbA\left( S_{ref},S_{alt},R \right)=dbA\left( S_{alt},R \right)-dbA\left( S_{ref},R \right)$

$S_{ref}$, $S_{alt}$ represent the reference and alternate sequences, respectively. Here, if any $P_{dbA}\left( S,R \right)<0.1$ then the corresponding $dbA\left( S,R \right)$ will be replaced by $0$. Negative $dbA$ values are also replaced by $0$ because no binding for background sequences is assumed. $\delta dbA$ measures the difference between the reference and the alternate sequences. There can be altogether three different scenarios in calculation of shifted differential binding affinity, with each case inferring a different result. In first case, if $\delta dbA>0$, then there is an increase in binding affinity of the alternate sequence as compared to that of the reference sequence. In other words, it may indicate creation of a new binding site for the TF or an increasing binding affinity for an existing weak binding site. In second case, if $\delta dbA<0$, then an existing binding site is disrupted or destroyed by the variant. In third case, if both reference and alternate $dbA$ are zero then $\delta dbA$ will also be zero, which means the sequence variant does not have an impact on TF binding affinity. In the new package, BayesPI-BAR is reimplemented in Python with an efficient parallel computation algorithm instead of original slow R scripts.

**2. BayesPI-BAR2 Package architecture**

BayesPI-BAR2 package contains a set of command line tools written in Python 2, which can be executed manually step by step. Alternatively, the whole pipeline can be executed automatically by a script *bayespi_bar2_pipeline.py*. The package reads files with simple text-based tab separated value (TSV) format. Each command line tool corresponds to a step of the pipeline that is described in Materials and Methods section. It runs in following order:

1. *differential_expression.py*: identifying a set of differentially expressed genes. Input: two sets of RNA-Seq gene expression data files, one for patients and the other for normal controls; the length of each gene in the reference genome must be provided. Output: a list of differentially expressed genes.
2. *gene_regions.py*: selecting DNA regions near TSS of differentially expressed genes. Input: a list of differentially expressed genes and a gene annotation file (GTF format). Output: a BED file with selected regions of interest.
3. *mussd.py*: running MuSSD algorithm to find patient mutation hot regions. Input: a Variant Call Format (VCF) file with somatic mutations for each patient, reference genome, regions of interest from the step 2. Optionally, VCF files with germline variants can be provided as well, in which case they will be used to generate personal reference sequences for every patient. Output: text files with the information of patient mutation blocks; for each patient mutation block, it creates a BED file which contains mutation location, a FASTA file with reference sequence, a FASTA file with mutated alternate sequences of each patient, and a TSV file listing all mutations in the block; finally, a summary information of all blocks, mutations, and patients.
4. *highly_mutated_blocks.py*: identifying significantly mutated patient mutation blocks based on random expectation by chance. This is an additional filter if there are too many patient mutation blocks recovered by MuSSD. Input: results from step 3. Output: a name list of highly mutated patient mutation blocks.
5. *bayespi_bar.py*: a new implementation of BayesPI-BAR algorithm. Input: reference/alternate sequence pairs (a pair of FASTA files); a set of position weight matrices (PWMs) for TFs of interest (a default set of 1772 PWMs from (Kheradpour and Kellis, 2014) [is included in the package), which are in BayesPI MLP format. Output: a table of $\delta dbA$](#_ENREF_1) scores for each PWM in every patient-specific mutation block.
6. *choose_background_parameters.py*: computing background mutation model. Input: results from *mussd.py*, the regions of interest, the reference genome, and a set of PWMs that was used in step 5. Optionally, a mutation signature file can be provided, which specifies the probability for mutations in each possible k-mer. If it is provided, the background mutations will be generated according to the given probability distribution; otherwise all possible mutations will have equal probabilities. Another option for background generation is to directly provide a set of background mutations, which normally will be derived from tumor samples. If these mutations are given, they will be randomly sampled to generate background mutation blocks. Only mutations located inside regions of interest are used. Output: creates a shell script that calls *bayespi_bar.py* to compute $\delta dbA$ scores in random background model; after executing the shell script, a table of $\delta dbA$ scores for each PWM in background model is produced.
7. *affinity_change_significance_test.py*: testing significance of TF binding affinity changes between the patient blocks and the background mutation model. Input: a pair of *bayespi_bar.py* results (one for a patient mutation block, and the other for a background model). Output: a TSV file containing a table of significantly affected TFs in the patient block.
8. *filter_results_by_gene_expression.py*: removing predicted TFs with very low gene expression. Input: a table of significantly affected TFs and their gene expression file. Output: a table of remaining TFs after the filtration.
9. *plot_result.py*: creating a summary plot of predicted mutation block such as Figure 3. Inputs: the background and foreground results from *bayespi_bar.py* and a table of significantly affected TFs. Output: a heat map in PNG format.

BayesPI-BAR2 package also provides simple error checking, logging, and it consolidates the pipeline parameters in one place. A description of command line options can be obtained by typing the *--help* option. All input and output data are text files, which are easy to inspect and modify (e.g. filter). The default script, *bayespi_bar2_pipeline.py*, runs the whole pipeline in the sequence. An evaluation of the genome-wide skin cancer data was performed by this script.

**3. User guide of BayesPI-BAR2 Python package**

To apply BayesPI-BAR2 package on real mutation dataset, following files are needed: one VCF file per patient containing somatic mutations (BayesPI-BAR2 accepts only SNVs), gene expression data for patients (e.g., RNA-Seq count files), normal control gene expression data, a reference genome FASTA file, and a gene annotation GTF file. Then, a user should provide the paths of input data and adjust pipeline parameters at the beginning of the pipeline script file, *bayespi_bar2_pipeline.py*. No proficiency in Python programming is needed for modifying the script, because it has a simple structure and is commented in all places that may need to be modified. Once the pipeline file is ready, it can be run by typing “*python bayespi_bar2_pipeline.py*” in the terminal. The pipeline will write a text log to track the progress and errors (e.g., missing input data). The package is designed to be robust against interruptions: for example, if the pipeline stops at some point, it can be restarted again from the place where it had been stopped. In BayesPI-BAR2 Python package, there is also a demo script to reproduce all results from analyzing skin cancer data. First, a script file *get_and_preprocess_data.py* will automatically download the skin cancer dataset, the reference genome data, and prepare the input files. Then, the demo script can be run without any modifications. More detailed user guide, including usage information for each tool in the package, is available at the website <http://folk.uio.no/junbaiw/BayesPI-BAR2/>.

**Supplementary Results**

**1. Supplementary Results of Skin cancer study**

Among genes with mutation blocks, there are two which were previously reported having regulatory mutations in melanoma: *SHDH* (Weinhold et al., 2014) [and](#_ENREF_5) *NDUFB9* (Poulos et al., 2015)[. However, occurrence rates of mutations in promoters of these two genes are low in the current dataset (12 patients, <5%), we did not investigate these genes further.](#_ENREF_2)

**Supplementary Figures**

**Supplementary Figure 1.** **Results of new BayesPI-BAR2 package - TFs significantly affected by skin cancer somatic mutations in the TERT promoter mutation block**. The heatmap displays the distribution of predicted TF binding effects of TERT promoter somatic SNVs across 58 skin cancer patients. The columns represent patients, the rows represent predicted significantly affected TFs, and the color represents the binding effect size. Reduced binding is shown in blue and increased binding in orange. The color shade represents the log_10_-scaled fraction of background δdbA values which are more extreme than the observed δdbA of a patient, which is an indication of effect size. The darker cells the larger effect. Some TFs are represented by multiple PWM models, their instances are indicated by a number in parentheses. Only significantly affected TFs (Wilcoxon rank-sum test P value < 0.001 after Bonferroni correction) are shown. The following TFs belong to the ETS family (Gutierrez-Hartmann et al., 2007): GABPα, ELF4, ETV5, ELK1, ETS1, SPI1, SPIB. Here, background mutation signature in BayesPI-BAR2 is generated based on "signature 7" (UV light damage, skin cancers) from COSMIC (https://cancer.sanger.ac.uk/cosmic/signatures), which with large numbers of CC>TT dinucleotide mutations at dipyrimidines.


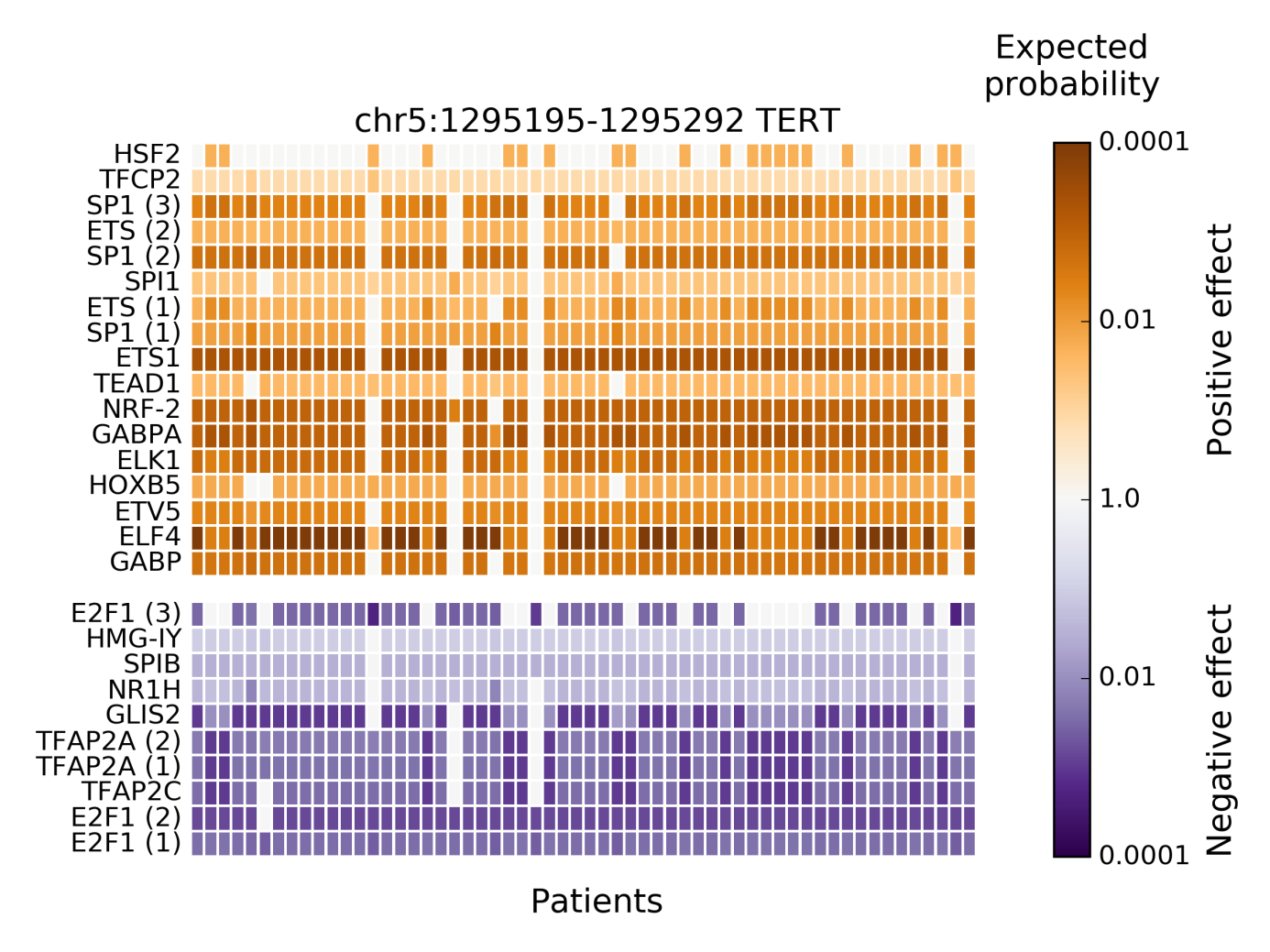


**Supplementary Figure 2.** **Results of new BayesPI-BAR2 package - TFs significantly affected by skin cancer somatic mutations in the TERT promoter mutation block**. The heatmap displays the distribution of predicted TF binding effects of TERT promoter somatic SNVs across 58 skin cancer patients. The columns represent patients, the rows represent predicted significantly affected TFs, and the color represents the binding effect size. Reduced binding is shown in blue and increased binding in orange. The color shade represents the log_10_-scaled fraction of background δdbA values which are more extreme than the observed δdbA of a patient, which is an indication of effect size. The darker cells the larger effect. Some TFs are represented by multiple PWM models, their instances are indicated by a number in parentheses. Only significantly affected TFs (Wilcoxon rank-sum test P value < 0.001 after Bonferroni correction) are shown. The following TFs belong to the ETS family (Gutierrez-Hartmann et al., 2007): GABPα, ELF4, ETV5, ELK1, ETS1, SPI1, SPIB. Here, the uniform background mutation signature is used.


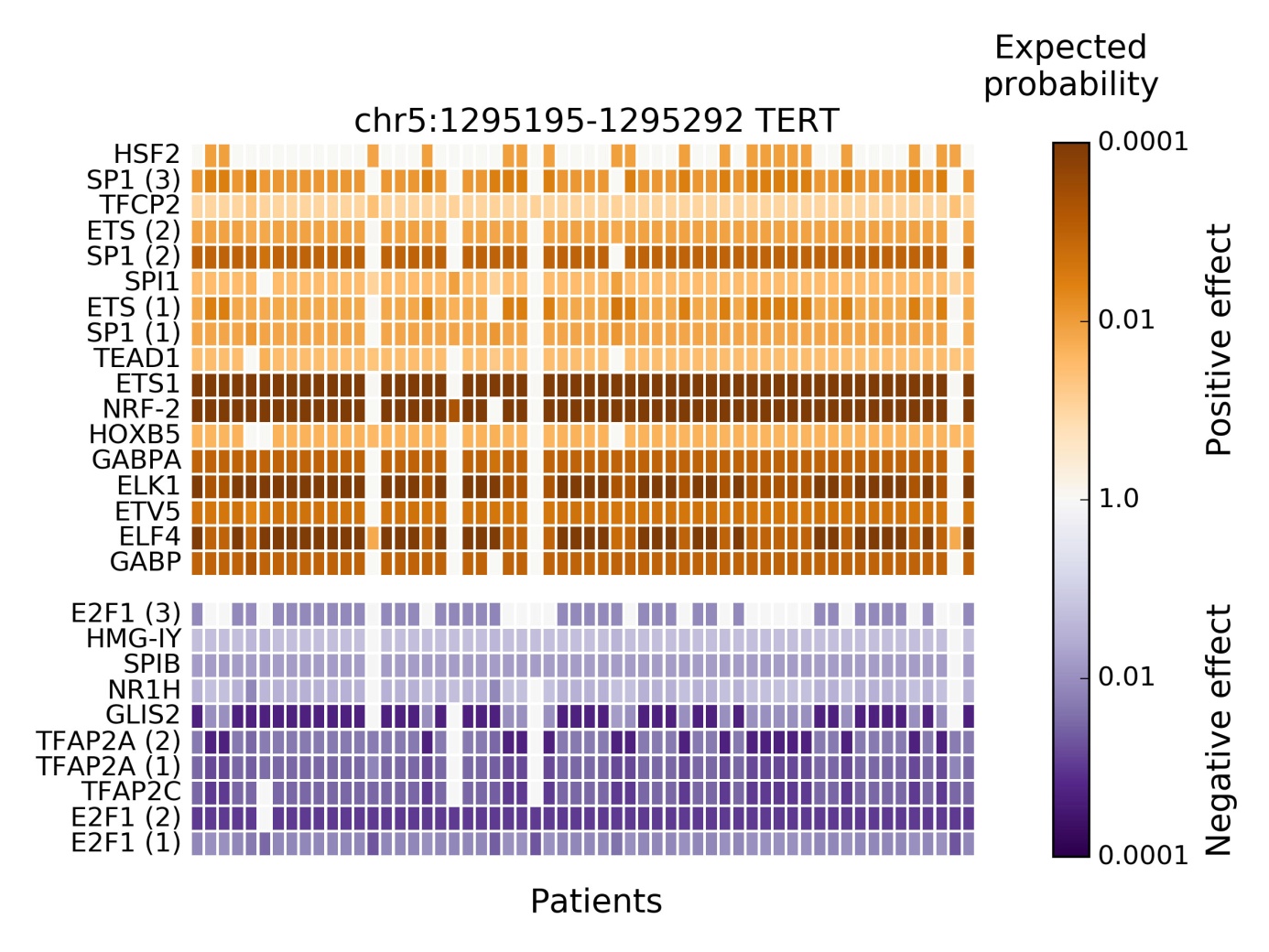


**References**

Kheradpour, P., and Kellis, M. (2014). Systematic discovery and characterization of regulatory motifs in ENCODE TF binding experiments. *Nucleic Acids Res* 42**,** 2976-2987.

Poulos, R.C., Thoms, J.A., Shah, A., Beck, D., Pimanda, J.E., and Wong, J.W. (2015). Systematic Screening of Promoter Regions Pinpoints Functional Cis-Regulatory Mutations in a Cutaneous Melanoma Genome. *Mol Cancer Res* 13**,** 1218-1226.

Wang, J., and Batmanov, K. (2015). BayesPI-BAR: a new biophysical model for characterization of regulatory sequence variations. *Nucleic Acids Res* 43**,** e147.

Wang, J., Malecka, A., Trøenand, G., and Delabie, J. (2015). Comprehensive genome-wide transcription factor analysis reveals that a combination of high affinity and low affinity DNA binding is needed for human gene regulation *BMC Genomics* 16 (Suppl 7):S12.

Weinhold, N., Jacobsen, A., Schultz, N., Sander, C., and Lee, W. (2014). Genome-wide analysis of noncoding regulatory mutations in cancer. *Nat Genet* 46**,** 1160-1165.
